# Association of Quantified Costal Cartilage Calcification and Long-Term Cumulative Blood Glucose Exposure: The Multi-Ethnic Study of Atherosclerosis

**DOI:** 10.3389/fendo.2021.785957

**Published:** 2021-12-13

**Authors:** Mahsima Shabani, Farhad Pishgar, Sepehr Akhtarkhavari, Thiago Quinaglia, Matthew J. Budoff, David A. Bluemke, Graham R. Barr, Wendy S. Post, Colin O. Wu, Armin Arbab-Zadeh, Aniket Sidhaye, João A. C. Lima, Shadpour Demehri

**Affiliations:** ^1^Department of Cardiology, School of Medicine, Johns Hopkins University, Baltimore, MD, United States; ^2^Department of Radiology and Radiological Science, School of Medicine, Johns Hopkins University, Baltimore, MD, United States; ^3^Lundquist Institute, Harbor-University of California, Los Angeles (UCLA) Medical Center, Torrance, CA, United States; ^4^Department of Radiology, University of Wisconsin-Madison School of Medicine and Public Health, Madison, WI, United States; ^5^Department of Medicine, Columbia University Medical Center, New York, NY, United States; ^6^Office of Biostatistics Research, National Heart, Lung, and Blood Institute, National Institutes of Health, Bethesda, MD, United States; ^7^Department of Endocrinology, School of Medicine, Johns Hopkins University, Baltimore, MD, United States

**Keywords:** calcium score, glucose, cumulative, diabetes mellitus, marker, cartilage, soft tissue

## Abstract

**Aims:**

Anecdotal reports have suggested increased soft tissue calcification in individuals with long-term exposures to high blood glucose. The association of costal cartilage calcification (CCC), a reliably quantifiable marker obtainable from non-contrast cardiac computed tomography (CT) with cumulative fasting blood glucose (FBG) exposure, is unknown. In this study, we aimed to determine the association between quantified CCC and cumulative glucose exposure using non-contrast coronary artery calcium (CAC) scoring computed tomography (CT) images in the Multi-Ethnic Study of Atherosclerosis (MESA).

**Methods:**

The volume of bilateral CCC was quantified in high-density pixels (threshold of Hounsfield Unit>180) using the CAC scoring CT images acquired in the 5^th^ MESA exam. Prior long-term cumulative exposure to FBG was calculated by area under the FBG-time curve over ten years before the time of the CT exam.

**Results:**

A total of 2,305 participants (mean age: 69, female/male: 1.3) were included in this study. The median CCC volume was lower in females than males (1158 mm^3^ [IQR: 1751] vs. 3054 mm^3^ [3851], p<0.001). In cross-sectional analysis, quantified CCC was associated with FBG (9% increase per SD) and HbA1c (7% increase per SD) at the CT exam only in female participants after adjustment for age, race, BMI, and glomerular filtration rate. Only in female participants, quantified CCC was also associated with prior cumulative FBG (3% increase per decile change). In the subgroup of females with zero CAC scores, the adjusted CCC was still associated with FBG (13% increase per SD) at the time of CT exam and with prior cumulative FBG exposure (4% increase per decile change) before the CT exam.

**Conclusions:**

The CCC, a reliably quantified marker in non-contrast cardiac CT, is associated with 10-year cumulative FBG exposure only in female participants, even those with zero CAC.

## Introduction

High fasting blood glucose (FBG) is the fourth modifiable risk factor and second metabolic risk factor accountable for the higher disability-adjusted life-years globally (1). Clinically occult type 2 diabetes mellitus (DM) and associated long-term high cumulative FBG exposure commonly exists years prior to the initial diagnosis of DM (2). Therefore, DM complications can be commonly detected at the time of initial DM clinical diagnosis (3). Uncertain duration of exposure to high FBG, a modifiable risk factor for DM complications, has urged investigators to identify reliable, affordable, and easily obtainable markers for long-term cumulative exposure to FBG that can be implemented as a screening tool for earlier diagnosis of DM and its associated complications in at-risk population (4). Hemoglobin A1c (HbA1c) is currently the most widely used serum marker for prior cumulative FBG exposure, which only reflects the 2-3 months prior to the measurement and is associated with coronary artery calcification (CAC), even in individuals without the clinical diagnosis of type 2 DM (5, 6). This widely used marker also has other pitfalls, such as underestimation of the exposure to high blood glucose due to the lower lifespan of red blood cells in hyperglycemic status (7). In addition, disorders such as iron deficiency anemia which affect red blood cells turnover can negatively impact the interpretation of HbA1c (8).

In addition to the known association between DM and the extent of vascular calcifications such as CAC, anecdotal reports have strongly and repeatedly suggested an association between DM and extensive nonvascular soft tissue calcifications. However, only a few studies have systematically investigated such association between musculoskeletal soft tissue calcifications and DM (9–12). For instance, in a cross-sectional observational study, calcific tendinitis of the shoulder rotator cuff has been associated with the presence of DM (13). Similarly, age- and serum calcium level-independent association of DM with calcific shoulder periarthritis has been reported, particularly in those subjects with longstanding and poorly controlled DM (9). DM has also been suggested as an independent risk factor for the ossification of longitudinal ligament of spine (11), and diffuse idiopathic skeletal hyperostosis (10, 14).

Calcification of costal cartilage (CCC) can be easily and reliably quantified using conventional non-contrast chest computed tomography (CT) images, including non-contrast cardiac CT images acquired for Coronary Artery Calcium (CAC) scoring (15). CCC has been primarily considered an age-related process with distinct patterns according to sex (16). There are only a few scattered reports of extensive CCC in metabolic and endocrine disorders such as hypo- or hyperthyroidism, acromegaly, rickets, adrenogenital syndrome, Keutel syndrome, and abnormal hematologic syndromes such as porphyria (17–21). In the adult population, there have also been reports of extensive CCC in hematologic and local chest wall malignancies, chronic kidney disease, and warfarin therapy (17, 18). However, due to its overall asymptomatic nature, few prior works have attempted to reliably quantify CCC using non-contrast chest CT images. Therefore, little is known about the association between quantified CCC and long-term prior cumulative blood glucose exposure.

Since most individuals that undergo CAC scoring have an intermediate risk of cardiovascular disease (22), a considerable proportion of them are expected to have abnormal blood glucose for many years prior to the CT examination (23). Therefore, quantification of CCC may provide a clinically applicable, reliable, and easily obtainable marker for prior cumulative blood glucose exposure in this at-risk population. It can be easily quantified from the same CT obtained for CAC scoring without additional cost or radiation exposure. We, therefore, aimed to investigate the association of quantified CCC with cumulative FBG exposure in a cohort of adult participants stratified according to sex. We further evaluated this association in the specific subgroup of participants with zero CAC score to find any potential value of CCC quantification when there is no coronary calcification detected in the same CT examination.

## Methods

### Participant Selection

The MESA cohort at exam 5 consisted of 3,442 participants in the age range of 53 to 94 years who were recruited from six US centers (Wake Forest University, Winston-Salem, NC; Columbia University, New York, NY; Johns Hopkins University, Baltimore, MD; University of Minnesota, Minneapolis-St. Paul, MN; Northwestern University, Chicago, IL; UCLA, Los Angeles, CA) with four ethnic backgrounds: White, African American, Asian American, and Hispanic. Participants were free of clinical cardiovascular disease (CVD) at the baseline MESA exam. The details of the MESA study design have been published previously (24). The MESArthritis study is an ancillary retrospective study of the MESA cohort to investigate the association of CT-derived soft tissue biomarkers with cardiovascular and metabolic risk factors and clinical outcomes (25). Previous reports of the MESArthritis study used the whole-chest CT scans of the MESA participants in exam 5; however, we have used the cardiac CT scans for our analysis (25). Available non-contrast cardiac CT images from 3,305 participants acquired in the 5^th^ MESA exam (2010-12) were analyzed. Participants with CT images identified as unevaluable by the readers (due to artifacts in the target field [including metal wire or plates due to previous surgery, cardiac pacemaker, or breast implants] or loss of required field of view (FOV) during reconstruction) were excluded. The demographic and clinical characteristics of participants were collected from the MESA database of the fifth MESA exam (2010-12), and all the preceding exams (exam one [2000-02], exam two [2002-04], exam three [2004-05], and exam four [2005-07]).

Institutional review boards at each of the six field centers recruited in the MESA study approved the study protocol. All participants gave written informed consent as part of the main study.

### Non-Contrast Cardiac Scan

The standardized cardiac CT protocol for the MESA study and the details of image reconstruction has been previously published (26). The non-contrast cardiac CT scans were performed by cardiac-gated electron-beam CT scanners: Toshiba One (320 slices, Toshiba Medical Systems, Japan), Siemens 64 (Siemens, Erlangen, Germany), Siemens Somatom Definition (Siemens, Erlangen, Germany), and General Electric VCT (64 slices, General Electric, Milwaukee, WI). All participants were scanned by certified technologists over phantoms of known physical calcium concentration. Images were reconstructed and analyzed for CAC score at the MESA CT reading center (Los Angeles Biomedical Research Institute at Harbor–UCLA in Torrance, California). CCC measurements were performed at the Johns Hopkins University, Baltimore, MD, USA.

### CCC Measurement

The most lateral concave line from the mid-sternal line was defined as the costochondral junction. In the axial view, the area medial to the junction, lateral to the cortex of sternum, and anterior to the hypodense lung air was considered the costal cartilage. The hyperdense tissue (HU > 180) lying within this area was defined as CCC. (**Figure S2**) The first pair of cartilages entirely visible in the superior end of the image FOV, usually 5^th^- 7^th^ rib, was selected for CCC quantification. The straight configuration and relatively parallel orientation of this pair to the axial CT plane compared to the ribs below facilitated measurements and increased their reproducibility (16, 27). Using the same infrastructure for CAC Volume score measurement in the Vitrea platform (Vitrea 7.11, Vital Images), the calcification score for each costal cartilage is calculated as the sum of calcified voxels with a predefined calcium threshold (28). A threshold of 180 HU was applied to separate calcifications from the surrounding soft tissue to prevent overestimation of calcifications compared to CAC (15).

A trained reader blinded to the risk factor profile of participants (a research fellow who was trained by a musculoskeletal radiologist with nine years of experience, and accomplished quantification of 50 test images with inter-reader reliability of >90% compared to the measurements of the radiologist) quantified the calcification. The reliability of the measurements was assessed by inter-reader agreement of 50 randomly selected images with a cardiovascular imaging research fellow with two years of experience.

### Statistical Analysis

CCC distribution within sex, age, race, and body-mass-index (BMI) categories was illustrated and compared within each category using violin plots, which show both the relevant summary statistics and the full distribution of data. The distribution of covariates in this study was presented as mean (standard deviation) or number (%) in different sex-specific quartiles of CCC. We assessed the missingness of covariates using the Little test (test of missing completely at random). A two-way intraclass correlation coefficient was calculated for the absolute inter-reader agreement of the CCC measurements.

In cross-sectional analysis, uni- and multivariate linear regression models adjusted for age, race, BMI, and glomerular filtration rate (GFR) were used to estimate the association between CCC and DM status, FBG (mg/dL), HbA1c (%), serum insulin (mU/L), oral hypoglycemic agent use, insulin resistance index of HOMA-IR (calculated as FBG × insulin/405) and confirmed diagnosis of metabolic syndrome at the time of CT, stratified by sex. For linear regression analyses, log transformation of the CCC was used as the dependent variable since the original CCC was not normally distributed in this study participants (Shapiro-Wilk test p-value <0.001).

**Figure 1:**
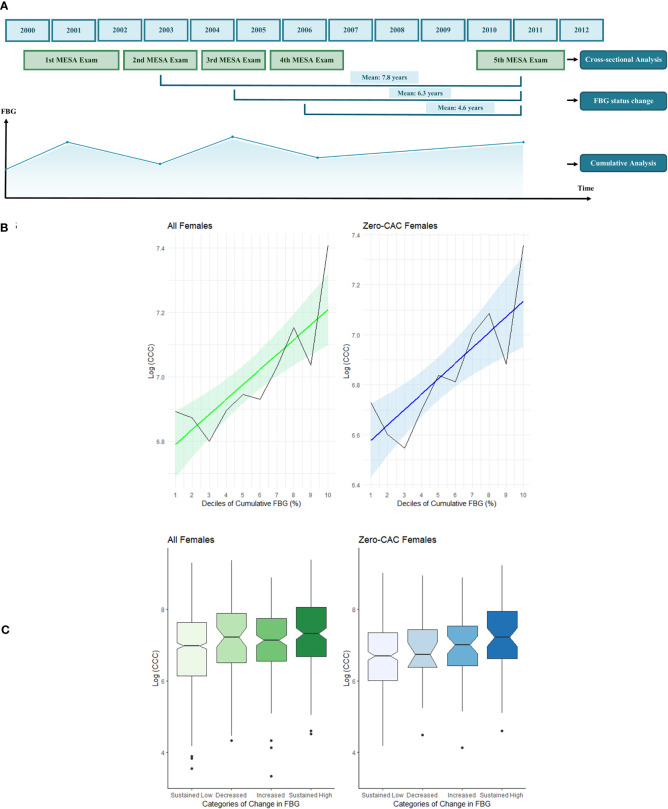
Timeline of the analyses of this study with regards to the main MESA exams **(A)**. The association of CCC with cumulative FBG exposure **(B)** and temporal change in FBG in mean follow-up interval of 4.6 years before the CT exam **(C)**. The cumulative analysis was performed using the area-under-curve of the five time points of FBG measurement during MESA exams 1 to 5. A sample FBG-time curve is illustrated. The graphs on left (green) illustrates the associations in total female participants. The graphs on right (blue) illustrates the associations in female participants with zero CAC score. The black lines show the bar chart of median CCC in deciles of cumulative FBG exposure. The colored line depicts the linear regression between CCC and deciles of cumulative FBG exposure. The colored areas fill in the 95% CI of the regression line. The notched bar charts show that participants with sustained high FBG values at both exams had higher CCC scores than participants with sustained low FBG. The CCC values provided in this figure are the log transformation of the original CCC.

In the cumulative analysis, all the available data from the MESArthritis participants between the first MESA exam (2000-02) and the fifth MESA exam (2010-12) were extracted. We used area-under-curve (AUC) of the FBG - time curve over ten years before CT to determine the long-term cumulative exposure to FBG (**Figure 1**). The cumulative FBG exposure was reported in milligram per deciliter multiplied by t in years, then the change in log (CCC) was illustrated within deciles of cumulative FBG exposure.

To further evaluate if the temporal changes in FBG status in different intervals can also affect the CCC score, we categorized the participants based on the value of FBG at CT (exam 5) and either of the previous exams (exam 2, 3, or 4) (**Figure 1**). Participants with repeated clinically normal FBG values (<100 mg/dL) were classified as “Sustained low”, and those with repeated values of above normal were “Sustained High”. Participants with normal FBG status (i.e., FBG values<100 mg/dL) at CT and above normal values in the previous exam were defined as “Decreased” and those with high FBG values (i.e., FBG values>100 mg/dL) at CT and normal values in the previous exam were defined as “Increased”.

Statistical analyses were performed using Rstudio programming software (version 1.3.1093). A p-value of 0.05 was used to determine statistical significance in linear regression models.

## Results

### Participant Characteristics and CCC Distribution

**Figure 2:**
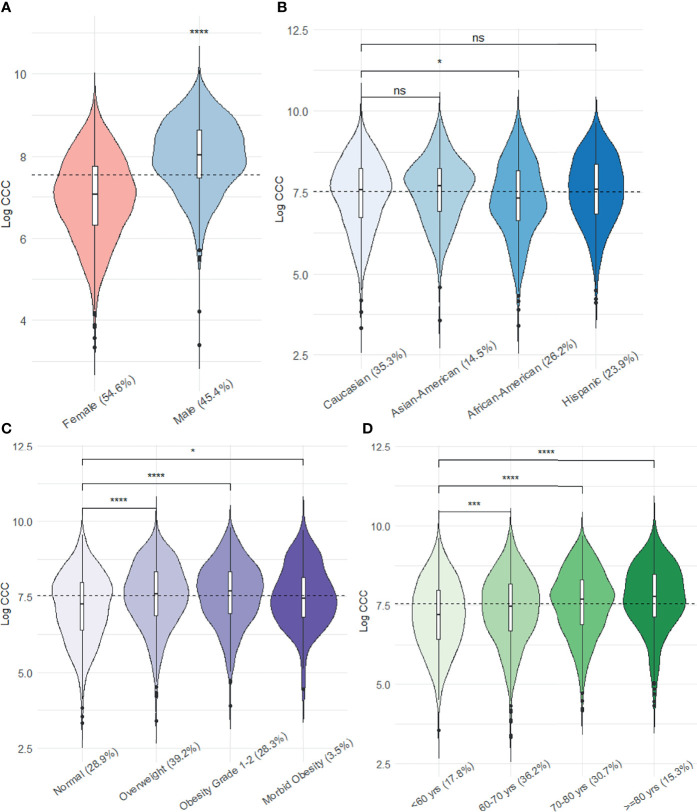
Sex- **(A)**, age- **(B)**, race- **(C)**, and BMI **(D)** category-specific distribution of the log transformed CCC. (The dashed line shows the median CCC of all participants; ns: p value > 0.05, *: p value <0.05, ***: p value <0.001, ****: p value <0.0001).

After excluding images with artifacts in the target field or incompliant FOV (n=743), CCC was measured in 2,562 participants. Participants with unavailable serum glucose or history of coronary artery bypass surgery and cancer before the CT were also excluded (n=257), and therefore, 2,305 participants were included in this analysis (**Figure S1**). The data were missed completely at random (p=0.353). Participants with a missing main independent variable of each analysis were excluded accordingly. At the time of the fifth MESA CT examination (2010-12), the participants had a mean age of 69 years (53 to 94 years); 54.6% were female, 35.3% White, 26.2% African American, 14.5% Asian American, and 23.9% were Hispanic (**Figure 2**). The median volume score of CCC was 1158 mm^3^ in females (Inter-Quartile Range [IQR]: 1751) and 3054 mm^3^ in males (IQR: 3851). Inter-reader reliability of CCC measurements was 88.7 (95% CI: 74.4-95.0).

A total of 439 participants had been diagnosed with DM prior to the CT acquisition. Of all 2305 participants, 755 had a CAC score of zero (68% female).

The CCC was significantly lower in African American participants than non-Hispanic Caucasians. Overweight (BMI of 25-30), obese (BMI of 30-40), and morbidly obese (BMI > 40) participants had higher CCC than participants with normal BMI. Participants in older age groups (60-70, 70-80, and >=80) had higher CCC than those younger than 60 years of age (**Figure 2**).

There were significant differences between age (p<0.001 both in females and males), BMI (p<0.001 in females and males), GFR (p<0.001 in females and males), as well as serum FBG (p<0.001 in females and males), HbA1c (p=0.003 in females, p=0.017 in males), insulin (p=0.002 in females, p=0.002 in males), DM status (p<0.001 in females, p=0.026 in males), use of oral hypoglycemic agent use (p=0.002 in females, p<0.001 in males) and insulin resistance index (p<0.001 in females and males) of participants in different quartiles of CCC in both female and male groups (**Table 1**).

**Table 1:** Distribution of the selected variables within quartiles of CCC in males and females separately.

	Participants	1^st^ Quartile	2^nd^ Quartile	3^rd^ Quartile	4^th^ Quartile	p value
**Female Sex**	All	492 (86.0%)	371 (64.0%)	252 (43.8%)	143 (24.8%)	**<0.001**
**CCC**	Female	458 (220)	1298 (283)	2652 (517)	5495 (1706)	
	Male	565 (192)	1326 (307)	2708 (538)	7045 (2836)	
**Number of participants**	Female	316	313	313	316	
	Male	262	260	263	262	
**Age (year)**	Female	66.6 (8.8)	67.8 (8.8)	70.0 (9.2)	71.9 (9.4)	**<0.001**
	Male	66.9 (9.0)	68.8 (9.4)	68.3 (9.2)	71.4 (9.0)	**<0.001**
**Race**						
White	Female	115 (36.4%)	101 (32.3%)	106 (33.9%)	109 (34.5%)	0.076
Chinese American		39 (12.3%)	39 (12.5%)	40 (12.8%)	49 (15.5%)	
African American		101 (32.0%)	101 (32.3%)	85 (27.2%)	70 (22.2%)	
Hispanic		61 (19.3%)	72 (23.0%)	82 (26.2%)	88 (27.8%)	
White	Male	94 (35.9%)	108 (41.5%)	91 (34.6%)	91 (34.7%)	**0.025**
Chinese American		36 (13.7%)	47 (18.1%)	51 (19.4%)	33 (12.6%)	
African American		77 (29.4%)	45 (17.3%)	62 (23.6%)	64 (24.4%)	
Hispanic		55 (21.0%)	60 (23.1%)	59 (22.4%)	74 (28.2%)	
**BMI (Kg/m^2^)**	Female	26.8 (5.7)	28.7 (6.1)	29.5 (6.0)	29.4 (6.3)	**<0.001**
	Male	26.3 (4.2)	27.5 (4.3)	28.3 (4.3)	29.3 (4.7)	**<0.001**
**Waist circumference (cm)**	Female	92.4 (14.1)	96.9 (15.7)	99.3 (15.2)	99.5 (15.3)	**<0.001**
	Male	96.2 (11.1)	99.2 (11.3)	101.3 (11.8)	104.2 (12.3)	**<0.001**
**SBP (mmHg)**	Female	121.3 (20.9)	124.9 (23.4)	127.1 (21.7)	130.4 (22.0)	**<0.001**
	Male	122.6 (18.2)	122.4 (21.1)	121.6 (17.9)	124.3 (18.4)	0.253
**DBP (mmHg)**	Female	65.8 (9.6)	67.1 (9.4)	66.4 (10.1)	67.0 (10.0)	0.313
	Male	73.1 (9.7)	70.8 (10.3)	71.3 (9.5)	71.0 (9.2)	**0.039**
**GFR (mL/min/1.73 m2)**	Female	66.7 (13.6)	66.4 (14.9)	66.5 (15.0)	64.2 (15.6)	**<0.001**
	Male	70.1 (11.8)	70.0 (14.2)	68.6 (15.6)	68.7 (14.8)	**<0.001**
**DM Status**						
Normoglycemic	Female	242 (76.8%)	200 (63.9%)	187 (59.9%)	178 (56.7%)	**<0.001**
Pre-diabetic		39 (12.4%)	59 (18.8%)	57 (18.3%)	58 (18.5%)	
Diabetic		34 (10.8%)	54 (17.3%)	68 (21.8%)	78 (24.8%)	
Normoglycemic	Male	160 (61.3%)	137 (52.7%)	141 (54.0%)	121 (46.4%)	**0.026**
Pre-diabetic		63 (24.1%)	67 (25.8%)	73 (28.0%)	76 (29.1%)	
Diabetic		38 (14.6%)	56 (21.5%)	47 (18.0%)	64 (24.5%)	
**FBG (mg/dL)**	Female	96.0 (18.1)	97.3 (18.7)	102.9 (32.4)	104.3 (30.0)	**<0.001**
	Male	101.7 (28.3)	104.9 (26.0)	102.2 (22.5)	106.5 (25.2)	**<0.001**
**HbA1c (%)**	Female	5.8 (0.7)	5.9 (0.7)	6.0 (1.0)	6.1 (1.0)	**0.003**
	Male	5.8 (0.8)	6.0 (0.8)	5.9 (0.8)	6.0 (0.8)	**0.008**
**Serum Insulin (mU/L)**	Female	52.4 (33.6)	59.4 (38.8)	66.0 (49.9)	64.6 (43.6)	**0.001**
	Male	53.2 (59.1)	59.5 (44.5)	59.8 (41.6)	65.4 (52.3)	**0.001**
**HOMA-IR**	Female	12.8 (9.3)	14.9 (11.8)	17.8 (17.5)	17.7 (14.4)	**<0.001**
	Male	13.9 (19.4)	16.7 (17.6)	15.6 (13.0)	17.6 (14.0)	**<0.001**
**Oral hypoglycemic agent use**	Female	26 (8.2%)	40 (12.8%)	53 (16.9%)	58 (18.4%)	**<0.001**
	Male	23.0 (8.8%)	43.0 (16.5%)	38.0 (14.4%)	53.0 (20.2%)	**0.003**
**CAC number (Agatston score)**	Female	107.8 (259.1)	139.8 (388.6)	162.6 (388.4)	240.1 (479.0)	**<0.001**
	Male	253.3 (464.8)	348.5 (603.6)	340.9 (544.7)	525.5 (922.2)	**<0.001**
**Zero-CAC participants**	Female	154 (48.7%)	148 (47.3%)	113 (36.2%)	99 (31.4%)	**<0.001**
	Male	76 (29.1%)	63 (24.2%)	65 (24.8%)	37 (14.1%)	**<0.001**
**Metabolic Syndrome**	Female	82 (26.1%)	119 (38.1%)	140 (44.9%)	148 (47.1%)	**<0.001**
	Male	56 (21.5%)	68 (26.2%)	91 (34.9%)	110 (42.3%)	**<0.001**

Mean (SD) is used for quantitative variables, and number (percentage) is used for categorical variables.

CCC, costal cartilage calcification; BMI, body mass index; SBP, systolic blood pressure; DBP, diastolic blood pressure; FBG, fasting blood glucose; HOMA-IR, HOmeostatic Model Assessment Insulin Resistance; CAC, coronary artery calcification. p values in rows with significant difference (<0.05) of the selected variable in different quartiles of CCC are marked in bold.

### Association of Quantitative CCC and Fasting Blood Glucose

CCC was strongly associated with age (2.6% increase per each year [95%CI: 2.0-3.2%] in female, 1.5% [1.0-2.1%]) in male) and BMI (2.9% increase per each kg/m^2^ [2.3-3.6%] in female, 6.0% [4.8-7.1%] in male) (**Table 2**). After adjustment for age, race, BMI, and GFR, CCC was positively associated with FBG (9.4% increase per SD [4.1-15.0%]), HbA1c (7.2% increase per SD [1.0-12.7%]), serum insulin (7.2% increase per SD [1.0-15.0%]), and HOMA-IR (10.5% increase per SD [3.0-17.3%]) in females but not in males. Female participants using oral hypoglycemic agents had higher CCC values (24.6% increase [6.2-44.8%]) than those who did not use oral hypoglycemic agents. Furthermore, female participants with diagnosed DM had higher CCC (24.6% increase [8.3-44.8%]) compared to normoglycemic participants, and those with metabolic syndrome had higher CCC compared to those without (18.5% increase [5.1-32.3]) (**Table 2**).

**Table 2:** Sex-specific association of CCC with age, BMI, and indicators of DM, using linear regression models in total participants and those with zero CAC score.

	Female				Male			
	Beta (95%CI) p value				Beta (95%CI) p value			
	Crude	Adjusted Model 1	Adjusted Model 2	Adjusted Model 3	Crude	Adjusted Model 1	Adjusted Model 2	Adjusted Model 3
**All Participants**								
**Age (years) **	**0.03 (0.02 to 0.03) <0.001**				**0.01 (0.01 to 0.02) <0.001**			
**BMI (kg/m^2^) **	**0.03 (0.02 to 0.04) <0.001**	**0.03 (0.02 to 0.03) <0.001**			**0.05 (0.04 to 0.06) (<0.001) **	**0.06 (0.05 to 0.07) <0.001**		
**FBG (mg/dL) **	**0.14 (0.08 to 0.19) <0.001**	**0.13 (0.08 to 0.19) <0.001**	**0.09 (0.04 to 0.14) <0.001**	**0.09 (0.04 to 0.15) <0.001**	**0.05 (0.00 to 0.11) 0.047**	**0.06 (0.01 to 0.11) 0.024**	0.01 (-0.04 to 0.06) 0.584	0.00 (-0.05 to 0.05) 0.979
**HbA1c (%) **	**0.11 (0.06 to 0.17) <0.001**	**0.10 (0.05 to 0.16) <0.001**	**0.06 (0.01 to 0.11) 0.024**	**0.07 (0.01 to 0.12) 0.013**	0.05 (0.00 to 0.10) 0.081	0.04 (-0.01 to 0.10) 0.106	-0.01 (-0.07 to 0.04) 0.635	-0.03 (-0.08 to 0.02) 0.296
**HOMA-IR**	**0.17 (0.11 to 0.24) <0.001**	**0.20 (0.14 to 0.26) <0.001**	**0.11 (0.04 to 0.18) 0.002**	**0.10 (0.03 to 0.16) 0.005**	**0.07 (0.02 to 0.12) 0.009**	**0.08 (0.03 to 0.13) 0.001**	-0.01 (-0.07 to 0.04) 0.564	-0.03 (-0.08 to 0.02) 0.312
**Serum Insulin** **(mU/L) **	**0.14 (0.08 to 0.21) <0.001**	**0.18 (0.12 to 0.24) <0.001**	**0.08 (0.01 to 0.15) 0.030**	**0.07 (0.00 to 0.14) 0.038**	**0.07 (0.02 to 0.12) 0.004**	**0.09 (0.04 to 0.14) <0.001**	-0.01 (-0.06 to 0.04) 0.603	-0.02 (-0.07 to 0.03) 0.424
**DM Status**								
Normoglycemic	Ref.	Ref.	Ref.	Ref.	Ref.	Ref.	Ref.	Ref.
Pre-diabetic	**0.24 (0.09 to 0.39) 0.002**	**0.19 (0.05 to 0.34) 0.010**	0.10 (-0.05 to 0.25) 0.178	0.04 (-0.10 to 0.19) 0.576	**0.18 (0.06 to 0.30) 0.004**	**0.18 (0.06 to 0.30) 0.003**	0.08 (-0.03 to 0.20) 0.165	0.06 (-0.06 to 0.17) 0.346
Diabetic	**0.40 (0.25 to 0.54) <0.001**	**0.36 (0.22 to 0.50) <0.001**	**0.23 (0.08 to 0.37) 0.002**	**0.22 (0.08 to 0.37) 0.003**	**0.22 (0.09 to 0.36) 0.001**	**0.20 (0.07 to 0.34) 0.003**	0.05 (-0.08 to 0.19) 0.438	0.01 (-0.12 to 0.15) 0.841
**Oral Hypoglycemic agent use**	**0.34 (0.18 to 0.50) <0.001**	**0.31 (0.15 to 0.47) <0.001**	**0.21 (0.05 to 0.36) 0.009**	**0.22 (0.06 to 0.37) 0.006**	**0.25 (0.10 to 0.39) <0.001**	**0.22 (0.08 to 0.37) 0.003**	0.11 (-0.03 to 0.25) 0.139	0.07 (-0.07 to 0.21) 0.319
**Metabolic Syndrome**	**0.38 (0.26 to 0.49) <0.001**	**0.35 (0.24 to 0.46) <0.001**	**0.19 (0.07 to 0.31) 0.002**	**0.17 (0.05 to 0.28) 0.006**	**0.33 (0.21 to 0.44) <0.001**	**0.32 (0.21 to 0.43) <0.001**	0.06 (-0.07 to 0.18) 0.373	0.04 (-0.08 to 0.16) 0.524
**Participants with a zero CAC**								
**FBG (mg/dL) **	**0.26 (0.15 to 0.38) <0.001**	**0.24 (0.13 to 0.36) <0.001**	**0.18 (0.06 to 0.30) 0.003**	**0.13 (0.01 to 0.25) 0.031**	0.14 (-0.01 to 0.28) 0.073	0.14 (-0.00 to 0.29) 0.060	0.07 (-0.08 to 0.21) 0.379	0.04 (-0.10 to 0.19) 0.574
**HbA1c (%) **	**0.20 (0.09 to 0.31) <0.001**	**0.18 (0.07 to 0.29) 0.001**	**0.12 (0.01 to 0.23) 0.036**	0.09 (-0.02 to 0.20) 0.121	0.08 (-0.07 to 0.22) 0.296	0.08 (-0.07 to 0.22) 0.296	-0.00 (-0.14 to 0.14) 0.974	-0.01 (-0.15 to 0.13) 0.891
**HOMA-IR**	**0.23 (0.11 to 0.34) <0.001**	**0.25 (0.13 to 0.36) <0.001**	0.12 (-0.01 to 0.26) 0.075	0.09 (-0.04 to 0.23) 0.174	0.12 (-0.04 to 0.28) 0.151	0.13 (-0.03 to 0.30) 0.105	-0.05 (-0.23 to 0.12) 0.533	-0.10 (-0.27 to 0.07) 0.250
**Serum Insulin (mU/L) **	**0.14 (0.05 to 0.23) 0.002**	**0.17 (0.08 to 0.26) <0.001**	0.06 (-0.04 to 0.17) 0.360	0.05 (-0.06 to 0.17) 0.389	0.10 (-0.05 to 0.25) 0.188	0.11 (-0.03 to 0.26) 0.133	-0.07 (-0.23 to 0.09) 0.417	-0.10 (-0.26 to 0.06) 0.202
**DM Status**								
Normoglycemic	Ref.	Ref.	Ref.	Ref.	Ref.	Ref.	Ref.	Ref.
Pre-diabetic	**0.27 (0.03 to 0.52) 0.026**	0.23 (-0.01 to 0.47) 0.063	0.12 (-0.12 to 0.37) 0.311	0.03 (-0.21 to 0.28) 0.793	**0.33 (0.08 to 0.58) 0.011**	**0.33 (0.08 to 0.57) 0.010**	0.22 (-0.02 to 0.47) 0.070	0.15 (-0.09 to 0.039) 0.223
Diabetic	**0.43 (0.18 to 0.67) <0.001**	**0.39 (0.15 to 0.63) 0.002**	0.24 (-0.01 to 0.49) 0.063	0.18 (-0.07 to 0.43) 0.160	0.30 (-0.07 to 0.67) 0.108	0.30 (-0.07 to 0.67) 0.110	0.15 (-0.21 to 0.51) 0.402	0.14 (-0.22 to 0.49) 0.450
**Oral Hypoglycemic agent use**	**0.30 (0.02 to 0.59) 0.036**	0.27 (-0.01 to 0.55) 0.057	0.13 (-0.15 to 0.41) 0.365	0.11 (-0.17 to 0.39) 0.442	0.27 (-0.14 to 0.68) 0.201	0.25 (-0.16 to 0.6) 0.233	0.14 (-0.26 to 0.54) 0.481	0.13 (-0.27 to 0.52) 0.521
**Metabolic Syndrome**	**0.30 (0.12 to 0.49) 0.001**	**0.30 (0.12 to 0.48) 0.001**	0.14 (-0.05 to 0.34) 0.151	0.11 (-0.08 to 0.31) 0.245	**0.31 (0.05 to 0.56) 0.018**	**0.28 (0.02 to 0.54) 0.032**	-0.02 (-0.30 to 0.26) 0.874	-0.08 (-0.36 to 0.19) 0.546

Model 1 is adjusted for age; Model 2 is Model 1 plus adjustment for BMI, and race; Model 3 is Model 2 plus adjustment for GFR.

The log transformation of the original CCC was used for this analysis.

The beta coefficients reported for FBG, HbA1c, HOMA-IR, and serum insulin are per each SD increase of these variables.

FBG, fasting blood glucose; DM, diabetes mellitus; HOMA-IR, HOmeostatic Model Assessment Insulin Resistance; CAC, coronary artery calcification. Cells with significant associations are marked in bold.

CAC was associated with FBG, insulin, HOMA-IR, DM status, and oral hypoglycemic use in both sexes in comparison to CCC. (**Table S1**) In female participants with zero CAC score, CCC was also associated with FBG (13.9% increase per SD [1.0-28.4%]) (**Table 2**).

### Association of CCC and Cumulative FBG Exposure Before CT

Higher deciles of cumulative FBG exposure measured at five time points within ten years were associated with higher CCC in all females as well as in the subgroup of female participants with Zero CAC scores (**Figure 1**). Female participants with higher cumulative FBG exposure had higher CCC (3.0% increase per decile [1.0-5.1%]) (**Table 3**).

**Table 3:** The sex-specific association of CCC with cumulative FBG exposure from exam 1 to exam 5 using area under curve (AUC), and length of diagnosed DM and time points with high FBG, in total participants and those with zero CAC score.

Variable	Population	Beta (95% CI) p value			
		Crude	Adjusted Model 1	Adjusted Model 2	Adjusted Model 3
***Total Population***					
**Cumulative FBG exposure (per decile) **	***Male***	**0.03 (0.01 to 0.05) 0.001**	**0.03 (0.01 to 0.05) 0.001**	0.01 (-0.01 to 0.03) 0.175	0.01 (-0.00 to 0.03) 0.193
	***Female***	**0.05 (0.03 to 0.07) <0.001**	**0.04 (0.02 to 0.06) <0.001**	**0.02 (0.00 to 0.04) 0.013**	**0.03 (0.01 to 0.05) 0.017**
**Time-points with high FBG**	***Male***	**0.05 (0.02 to 0.08) <0.001**	**0.05 (0.02 to 0.08) 0.002**	0.02 (-0.01 to 0.05) 0.248	0.01 (-0.01 to 0.04) 0.320
	***Female***	**0.10 (0.06 to 0.13) <0.001**	**0.08 (0.05 to 0.11) <0.001**	**0.05 (0.02 to 0.08) 0.003**	**0.05 (0.02 to 0.09) 0.005**
**Length of diagnosed DM**					
**Never**	***Male***	Ref.	Ref.	Ref.	Ref.
**<5 yrs with DM**		0.15 (-0.06 to 0.36) 0.155	0.15 (-0.06 to 0.36) 0.165	0.03 (-0.17 to 0.23) 0.749	0.02 (-0.18 to 0.22) 0.817
**>5 yrs with DM**		**0.18 (0.00 to 0.35) 0.044**	0.14 (-0.03 to 0.31) 0.112	0.01 (-0.15 to 0.18) 0.876	0.01 (-0.16 to 0.18) 0.929
**Never**	***Female***	Ref.	Ref.	Ref.	Ref.
**<5 yrs with DM**		**0.23 (0.02 to 0.43) 0.028**	**0.27 (0.07 to 0.46) 0.009**	0.16 (-0.04 to 0.36) 0.119	0.14 (-0.06 to 0.34) 0.172
**>5 yrs with DM**		**0.48 (0.29 to 0.68) <0.001**	**0.39 (0.19 to 0.58) <0.001**	**0.29 (0.10 to 0.48) 0.003**	**0.31 (0.12 to 0.50) 0.002**
**Zero CAC Score**					
**Cumulative FBG exposure**	***Male***	**0.05 (0.01 to 0.10) 0.012**	**0.05 (0.01 to 0.10) 0.016**	0.03 (-0.01 to 0.07) 0.167	0.03 (-0.01 to 0.07) 0.171
	***Female***	**0.06 (0.03 to 0.09) <0.001**	**0.06 (0.03 to 0.09) <0.001**	**0.04 (0.01 to 0.07) 0.007**	**0.04 (0.01 to 0.07) 0.011**
**Time-points with high FBG**	***Male***	**0.07 (0.00 to 0.14) 0.044**	0.07 (-0.00 to 0.14) 0.056	0.02 (-0.04 to 0.09) 0.508	0.02 (-0.04 to 0.09) 0.491
	***Female***	**0.12 (0.06 to 0.17) <0.001**	**0.10 (0.04 to 0.16) <0.001**	0.07 (0.01 to 0.13) 0.029	**0.06 (0.00 to 0.12) 0.039**
**Length of diagnosed DM**					
**Never**	***Male***	Ref.	Ref.	Ref.	Ref.
**<5 yrs with DM**		0.26 (-0.23 to 0.75) 0.296	0.28 (-0.21 to 0.77) 0.258	0.13 (-0.34 to 0.59) 0.587	0.14 (-0.32 to 0.61) 0.537
**>5 yrs with DM**		0.12 (-0.43 to 0.67) 0.670	0.09 (-0.47 to 0.64) 0.756	-0.04 (-0.57 to 0.48) 0.873	-0.01 (-0.54 to 0.52) 0.959
**Never**	***Female***	Ref.	Ref.	Ref.	Ref.
**<5 yrs with DM**		**0.34 (0.03 to 0.66) 0.033**	**0.38 (0.07 to 0.69) 0.015**	0.27 (-0.04 to 0.58) 0.091	0.22 (-0.09 to 0.54) 0.170
**>5 yrs with DM**		**0.47 (0.09 to 0.85) 0.015**	0.34 (-0.03 to 0.72) 0.072	0.20 (-0.18 to 0.58) 0.300	0.21 (-0.17 to 0.59) 0.268

Model 1 is adjusted for age; Model 2 is Model 1 plus adjustment for race and BMI at exam 5; Model 3 is Model 2 plus adjustment for GFR.

Beta coefficients are reported per each decile change in cumulative FBG exposure.

The log transformation of the original CCC was used for this analysis.

FBG, fasting blood glucose; DM, diabetes mellitus; CAC, coronary artery calcification. Cells with significant associations are marked in bold.

Similarly, the number of time points with high FBG (FBG >100 mg/dL) was strongly associated with CCC in all females (5.1% increase per each additional time point [2.0-9.4%]) and those with zero-CAC (6.2% increase per each additional time point [1.0-12.7%]). Moreover, female participants with an over 5-year history of diagnosed DM had a 36.3% higher CCC score [12.7-64.9%] than participants with no DM. However, in female participants with a zero CAC score, CCC was not associated with the length of DM. The association of CAC and cumulative FBG exposure was shown in **Table S2**.

### Association of CCC and Temporal Changes in FBG Before CT

The mean interval between exam five (time of CT) and exams two, three, and four was 7.8, 6.3, and 4.6 years. Female participants with higher-than-normal FBG values at both exams had higher average CCC scores compared to participants with sustained low FBG (23.4% higher in the 4.6-year interval [95%CI: 7.2-43.3%], 29.7% higher in the 6.3-year interval [10.5-52.2%], and 23.4% higher in 7.8-year interval [6.2-44.8%]; **Figure 1** and **Table 4**). In comparison, both female and male participants with sustained high FBG in repeated exams had higher CAC scores than those with sustained low FBG (**Table S3**). However, in participants with zero CAC score, females with increased FBG levels had higher CCC compared to participants with sustained low FBG in the 4.6-year (32.3% higher [1.0-75.1%]) and 7.8-year (32.3% higher [1.0-75.1%]) intervals (**Table 4**).

**Table 4:** The association of CCC with categorized change in FBG status (normal (FBG=<100 mg/dL and above normal FBG >100 mg/dL) from exam 4 to exam 5 (mean interval of 4.6 yrs), from exam 3 to exam 5 (mean interval of 6.3 yrs), and exam 2 to exam 5 (mean interval of 7.8 yrs).

	Female				Male			
	Beta (95%CI) p value				Beta (95%CI) p value			
	Crude	Adjusted Model 1	Adjusted Model 2	Adjusted Model 3	Crude	Adjusted Model 1	Adjusted Model 2	Adjusted Model 3
**Exam 2 to Exam 5**								
***All Participants***								
Sustained low	Ref.	Ref.	Ref.	Ref.	Ref.	Ref.	Ref.	Ref.
Decreased	**0.25 (0.02 to 0.49) 0.032**	0.15 (-0.08 to 0.38) 0.190	0.06 (-0.16 to 0.29) 0.579	0.07 (-0.16 to 0.29) 0.568	0.04 (-0.20 to 0.28) 0.757	-0.03 (-0.27 to 0.20) 0.771	-0.08 (-0.30 to 0.15) 0.513	-0.09 (-0.32 to 0.14) 0.448
Increased	**0.21 (0.04 to 0.39) 0.016**	**0.18 (0.01 to 0.34) 0.041**	0.10 (-0.07 to 0.27) 0.231	0.12 (-0.05 to 0.29) 0.155	0.13 (-0.02 to 0.28) 0.091	0.14 (-0.01 to 0.29) 0.072	0.05 (-0.10 to 0.19) 0.511	0.04 (-0.10 to 0.19) 0.559
Sustained High	**0.39 (0.23 to 0.54) <0.001**	**0.32 (0.17 to 0.47) <0.001**	**0.21 (0.05 to 0.36) 0.008**	**0.21 (0.06 to 0.37) 0.006**	**0.25 (0.11 to 0.38) <0.001**	**0.23 (0.09 to 0.36) 0.001**	0.10 (-0.03 to 0.23) 0.142	0.09 (-0.04 to -0.23) 0.164
***Participants with a zero CAC***								
Sustained low	Ref.	Ref.	Ref.	Ref.	Ref.	Ref.	Ref.	Ref.
Decreased	0.37 (-0.02 to 0.76) 0.063	0.26 (-0.13 to 0.65) 0.195	0.18 (-0.21 to 5.33) 0.353	0.15 (-0.24 to 0.54) 0.448	0.35 (-0.12 to 0.82) 0.143	0.30 (-0.18 to 0.78) 0.214	0.25 (-0.20 to 0.71) 0.272	0.23 (-0.23 to 0.69) 0.329
Increased	**0.41 (0.14 to 0.69) 0.003**	**0.38 (0.10 to 0.65) 0.007**	**0.29 (0.02 to 0.56) 0.038**	**0.28 (0.01 to 0.56) 0.043**	0.28 (-0.01 to 0.57) 0.055	**0.29 (0.00 to 0.58) 0.047**	0.17 (-0.11 to 0.45) 0.240	0.16 (-0.12 to 0.44) 0.257
Sustained High	**0.40 (0.15 to 0.66) 0.002**	**0.34 (0.09 to 0.60) 0.007**	0.22 (-0.03 to 0.48) 0.089	0.21 (-0.05 to 0.47) 0.113	**0.39 (0.07 to 0.71) 0.017**	**0.38 (0.06 to 0.70) 0.020**	0.20 (-0.11 to 0.51) 0.209	0.21 (-0.10 to 0.52) 0.186
**Exam 3 to Exam 5**								
***All Participants***								
Sustained low	Ref.	Ref.	Ref.	Ref.	Ref.	Ref.	Ref.	Ref.
Decreased	0.20 (-0.05 to 0.46) 0.116	0.14 (-0.10 to 0.39) 0.253	0.04 (-0.21 to 0.28) 0.775	0.05 (-0.19 to 0.30) 0.674	0.22 (-0.02 to 0.46) 0.078	0.14 (-0.10 to 0.38) 0.260	0.06 (-0.17 to 0.29) 0.592	0.10 (-0.34 to 0.53) 0.661
Increased	0.17 (-0.00 to 0.34) 0.053	0.14 (-0.03 to 0.30) 0.103	0.06 (-0.10 to 0.22) 0.476	0.08 (-0.09 to 0.24) 0.367	**0.18 (0.04 to 0.33) 0.015**	**0.18 (0.04 to 0.33) 0.014**	0.09 (-0.05 to 0.23) 0.216	0.26 (-0.02 to 0.54) 0.070
Sustained High	**0.43 (0.28 to 0.59) <0.001**	**0.37 (0.22 to 0.53) <0.001**	**0.25 (0.09 to 0.41) 0.002**	**0.26 (0.10 to 0.42) 0.001**	**0.23 (0.09 to 0.37) 0.001**	**0.22 (0.08 to 0.35) 0.002**	0.09 (-0.05 to 0.22) 0.204	0.05 (-0.27 to 0.37) 0.754
***Participants with a zero CAC***								
Sustained low	Ref.	Ref.	Ref.	Ref.	Ref.	Ref.	Ref.	Ref.
Decreased	0.17 (-0.23 to 0.58) 0.401	0.14 (-0.26 to 0.54) 0.492	0.03 (-0.37 to 0.44) 0.875	0.00 (-0.40 to 0.41) 0.995	0.22 (-0.23 to 0.66) 0.336	0.18 (-0.27 to 0.62) 0.441	0.11 (-0.32 to 0.54) 0.607	0.10 (-0.34 to 0.53) 0.661
Increased	0.26 (-0.02 to 0.53) 0.065	0.23 (-0.04 to 0.50) 0.090	0.15 (-0.12 to 0.42) 0.282	0.16 (-0.12 to 0.43) 0.262	**0.41 (0.13 to 0.70) 0.005**	**0.42 (0.13 to 0.70) 0.004**	0.26 (-0.01 to 0.54) 0.062	0.26 (-0.02 to 0.54) 0.070
Sustained High	**0.51 (0.26 to 0.76) <0.001**	**0.45 (0.20 to 0.70) <0.001**	**0.33 (0.07 to 0.59) 0.013**	**0.30 (0.04 to 0.57) 0.023**	0.19 (-0.14 to 0.52) 0.253	0.18 (-0.14 to 0.51) 0.267	0.03 (-0.28 to 0.35) 0.830	0.05 (-0.27 to 0.37) 0.754
**Exam 4 to Exam 5**								
***All Participants***								
Sustained low	Ref.	Ref.	Ref.	Ref.	Ref.	Ref.	Ref.	Ref.
Decreased	0.17 (-0.04 to 0.38) 0.121	0.13 (-0.08 to 0.33) 0.226	0.05 (-0.16 to 0.25) 0.658	0.06 (-0.14 to 0.26) 0.574	-0.00 (-0.20 to 0.20) 0.993	-0.05 (-0.25 to 0.15) 0.643	-0.11 (-0.30 to 0.08) 0.265	-0.12 (-0.31 to 0.07) 0.227
Increased	**0.26 (0.06 to 0.45) 0.009**	**0.21 (0.02 to 0.40) 0.027**	0.12 (-0.06 to 0.31) 0.200	0.14 (-0.05 to 0.32) 0.153	0.13 (-0.03 to 0.30) 0.116	0.15 (-0.02 to 0.31) 0.080	0.08 (-0.08 to 0.24) 0.319	0.08 (-0.08 to 0.23) 0.348
Sustained High	**0.36 (0.21 to 0.51) <0.001**	**0.31 (0.17 to 0.45) <0.001**	**0.20 (0.06 to 0.35) 0.006**	**0.21 (0.07 to 0.36) 0.004**	**0.22 (0.09 to 0.35) <0.001**	**0.21 (0.08 to 0.34) 0.002**	0.06 (0.07 to 0.19) 0.336	0.05 (-0.07 to 0.19) 0.380
***Participants with a zero CAC***								
Sustained low	Ref.	Ref.	Ref.	Ref.	Ref.	Ref.	Ref.	Ref.
Decreased	0.21 (-0.13 to 0.55) 0.226	0.21 (-0.12 to 0.54) 0.213	0.16 (-0.17 to 0.50) 0.334	0.15 (-0.18 to 0.49) 0.357	-0.10 (-0.47 to 0.26) 0.581	-0.17 (-0.54 to 0.20) 0.377	-0.22 (-0.57 to 0.14) 0.230	-0.22 (-0.58 to 0.13) 0.220
Increased	**0.39 (0.11 to 0.67) 0.007**	**0.35 (0.07 to 0.62) 0.013**	0.27 (-0.00 to 0.55) 0.054	**0.28 (0.00 to 0.56) 0.048**	**0.36 (0.02 to 0.70) 0.037**	**0.37 (0.03 to 0.71) 0.030**	0.28 (-0.03 to 0.60) 0.080	0.29 (-0.03 to 0.61) 0.079
Sustained High	**0.44 (0.19 to 0.70) <0.001**	**0.40 (0.15 to 0.65) 0.002**	**0.29 (0.03 to 0.54) 0.030**	0.26 (-0.00 to 0.051)	0.25 (-0.04 to 0.53) 0.089	0.24 (-0.04 to 0.53) 0.092	0.04 (-0.24 to 0.32) 0.771	0.05 (-0.23 to 0.33) 0.744

Model 1 is adjusted for age; Model 2 is Model 1 plus race and BMI at exam 5.

The log transformation of the original CCC was used for this analysis. Cells with significant associations are marked in bold.

## Discussion

In this study, we have demonstrated that quantified CCC, obtained from the non-contrast cardiac CT scan images performed for CAC scoring, may be an indicator of prior blood glucose exposure only in female participants. The quantified CCC is associated with FBG, HbA1c, insulin, and insulin resistance index using cross-sectional analysis at the time of CT examination. Besides, quantified CCC is associated with the cumulative FBG exposure over ten years prior to the CT examination.

CT scan is an accurate and reliable tool for detecting and quantifying soft tissue calcifications (29) such as CCC. Quantified CCC is obtainable from any chest CT examination. For our hypothesis, we chose to only use CAC scoring non-contrast cardiac CT examinations, as patients who are referred for CAC scoring usually have an intermediate risk (7.5-20%) for atherosclerotic cardiovascular disease (ASCVD) (22). Therefore, DM prevalence or the risk of DM incidence in the future is substantial in these patients (23). The target population for CAC scoring CT examination is almost half of the US adult population between 40 to 75 years old (29). Thus, since a non-contrast cardiac CT scan imposes healthcare cost and radiation exposure to many screened patients in clinical practice, identifying a reliable and easily obtainable CT-based marker from these images at zero additional cost or radiation exposure would be cost-effective and tremendously beneficial to implement optimal secondary prevention measures for DM and its complications in these at-risk individuals.

Although calcification in cartilaginous tissue is a common age-related finding in the adult population particularly among the elderly, its clinical significance has not been thoroughly investigated. There have been several scattered case reports of premature or extensive CCC in patients with various metabolic disorders (18). Still, to date, no study has investigated the association of CCC with cumulative blood glucose exposure using a well-designed longitudinal database.

This study found a gender-specific association between CCC and prior cumulative blood glucose exposure up to ten years only in female participants. Prior works have shown higher CCC extents among male subjects and different CCC patterns between the two sexes, with a higher prevalence of marginal patterns in males vs. granular and central patterns in females (30). Similarly, reports have suggested various methods of sex identification by CCC, which further elute to the association between sex and CCC development and quantity (31). Such association between soft tissue calcification and sex is not limited to costal cartilages and other sex-dependent calcification patterns and mineralization pathways have also been suggested for vascular and brain calcifications (32, 33). Presence of an association between quantified CCC and prior FBG exposure only in female, may suggest a mediatory role for sex in soft tissue calcium depositions among subjects with impaired glucose homeostasis, which can be a subject for future investigations.

Although our proposed method for CCC quantification was not fully automated, it was easily implementable, did not require tremendous background experience for CT interpretation and the data was obtainable with high reliability.

We have also found that quantified CCC is associated with cumulative FBG exposure in females even those with zero CAC scores. Individuals with non-zero CAC scores are further classified based on the level of the CAC for CVD risk prediction and an indication of statin therapy (34). There is also a known association between the routinely quantified CAC scores and DM (35), and the value of CAC score improves the prognostication of incident coronary heart disease (CHD) in patients with DM (36). Therefore, demonstration of additional associations between quantified CCC and prior FBG exposure in nonzero CAC score subjects may be of limited clinical value. In contrast, subjects with CAC score of zero have lower risks of CHD and CVD their risk for 10-year all-cause mortality of about 1% (37). A large portion of subjects eligible for non-contrast CAC scoring cardiac CT in clinical practice have a CAC score of zero (50% in MESA participants) (35). Specifically, the younger participants (<55 years old) have a 70-90% prevalence of zero CAC score (38). Therefore, in female participants with zero CAC scores, the association between quantified CCC, obtainable from the same CT examination, and prior FBG exposure can provide an opportunity for implementing secondary preventive measures for DM and its complications.

The MESA cohort, with available imaging exams and multiple measurements of metabolic markers spread out through more than a decade provides an optimal platform for long-term cumulative analyses of prior FBG exposure. However, our study has several limitations. First, given that the primary MESA cohort has not been explicitly designed for the aim of this study. However, we tried to minimize this limitation using a relevant methodology regarding selection criteria and adjustment for possible confounders. Second, the limited number of participants who had available non-contrast cardiac CT and further stratification of them by sex resulted in a low sample size and limited the power of this study. Third, this study is also limited in differentiating between participants with different cumulative FBG levels before the baseline MESA exam. Fourth, although our CCC quantification method was reliable and easily obtainable in clinical practice, it has not been used or validated previously in the literature. We believe further validation using other cohorts and databases is warranted. Finally, previous studies have shown that calcium metabolism is impaired in diabetes type I and II (39–41). High blood glucose and advanced glycation end products impair the function of calcium-regulating hormones and organs involved in calcium metabolism, including the kidney, intestine, bone, and parathyroid glands (39). The levels of calcium-regulating hormones, osteoclastic cytokines, and serum and urine calcium were not available in most individuals, limiting the ability to address the mechanism underlying the association between CCC and FBG exposure in our study.

In conclusion, this is the first report of the association between CCC obtained from non-contrast cardiac CT and cumulative blood glucose exposure. This novel index may be an indicator of prior long-term cumulative glucose exposure in women, regardless of DM status and CAC score. Although the findings of this study were robust, we believe further validation of this association in prospective cohorts with a higher sample size and more specific follow-up data on incident DM and micro- and macrovascular complications of DM would be of potential value in diabetes care.

## Data Availability Statement

The datasets presented in this article are not readily available because upon MESA Coordination Center approval, the data will be available to the requesters. Requests to access the datasets should be directed to voodoo@u.washington.edu.

## Ethics Statement

The studies involving human participants were reviewed and approved by the recruitment centers' IRB committee, including the Johns Hopkins Medicine IRB committee. The patients/participants provided their written informed consent to participate in this study.

## Author Contributions

MS: Design, Analysis, and Interpretation of data; Drafting the article. FP: Analysis and Interpretation of data; Drafting the article. SA: Analysis of data; Drafting the article. TS: Conception and Design of study; Drafting the article. MB: Data Acquisition; Critical revision. DB: Design of study; Critical revision. GB: Data Acquisition; Critical revision. WP: Data Acquisition; Critical revision. CW: Analysis and Interpretation of data; Critical revision. AA-Z: Design of study and interpretation of data; Critical revision. AS: Interpretation of data; Critical revision. JL: Conception and Design of study and interpretation of data; Critical revision. SD: Conception and Design of study and interpretation of data; Critical revision. All authors contributed to the article and approved the submitted version.

## Funding

This research was supported by contracts 75N92020D00001, HHSN268201500003I, N01-HC-95159, 75N92020D00005, N01-HC-95160, 75N92020D00002, N01-HC-95161, 75N92020D00003, N01-HC-95162, 75N92020D00006, N01-HC-95163, 75N92020D00004, N01-HC-95164, 75N92020D00007, N01-HC-95165, N01-HC-95166, N01-HC-95167, N01-HC-95168 and N01-HC-95169 from the National Heart, Lung, and Blood Institute, and by grants UL1-TR-000040, UL1-TR-001079, and UL1-TR-001420 from the National Center for Advancing Translational Sciences (NCATS). This publication was developed under a STAR research assistance agreements, No. RD831697 (MESA Air) and RD-83830001 (MESA Air Next Stage), awarded by the U.S Environmental Protection Agency. It has not been formally reviewed by the EPA. The views expressed in this document are solely those of the authors and the EPA does not endorse any products or commercial services mentioned in this publication.

## Author Disclaimer

The views expressed in this manuscript are those of the authors and do not necessarily represent the views of the National Heart, Lung, and Blood Institute; the National Institutes of Health; or the U.S. Department of Health and Human Services.

## Conflict of Interest

The authors declare that the research was conducted in the absence of any commercial or financial relationships that could be construed as a potential conflict of interest.

The reviewer AS declared a shared affiliation with one of the authors, CW, to the handling editor at time of review.

## Publisher’s Note

All claims expressed in this article are solely those of the authors and do not necessarily represent those of their affiliated organizations, or those of the publisher, the editors and the reviewers. Any product that may be evaluated in this article, or claim that may be made by its manufacturer, is not guaranteed or endorsed by the publisher.
