# Supplementary material for: Association of Quantified Costal Cartilage Calcification and Long-Term Cumulative Blood Glucose Exposure: The Multi-Ethnic Study of Atherosclerosis
Source: Front Endocrinol (Lausanne). 2021 Dec 13;12:785957. doi: 10.3389/fendo.2021.785957 (PMC8711271; doi:10.3389/fendo.2021.785957)

**Supplementary material:**

Table S1. Sex-specific association of Log CAC with DM RFs and indicators using linear regression models in total population. *Model 1 is adjusted for age; Model 2 is Model 1 plus adjustment for BMI, and race. FBG, fasting blood glucose; DM, diabetes mellitus; HOMA-IR, HOmeostatic Model Assessment Insulin Resistance.*

|  | Female | | | Male | | |
| --- | --- | --- | --- | --- | --- | --- |
|  | **Beta (p value)** | | | Beta (p value) | | |
|  | **Crude** | **Adjusted 1** | **Adjusted 2** | **Crude** | **Adjusted 1** | Adjusted 2 |
| FBG (mg/dL) | **0.30 (0.16 to 0.44)**  **<0.001** | **0.28 (0.16 to 0.41)**  **<0.001** | **0.27 (0.14 to 0.40)**  **<0.001** | **0.30 (0.14 to 0.46)**  **<0.001** | **0.35 (0.20 to 0.49) <0.001** | 0.31 (0.16 to 0.46)  <0.001 |
| HbA1c (%) | **0.38 (0.24 to 0.51)**  **<0.001** | **0.33 (0.21 to 0.45)**  **<0.001** | **0.32 (0.19 to 0.44)**  **<0.001** | **0.33 (0.17 to 0.48)**  **<0.001** | **0.37 (0.21 to 0.52)**  **<0.001** | 0.33 (0.17 to 0.48) <0.001 |
| HOMA-IR | **0.19 (0.03 to 0.35)**  **0.020** | **0.31 (0.17 to 0.46)**  **<0.001** | **0.29 (0.13 to 0.45) <0.001** | **0.24 (0.09 to 0.40)**  **0.002** | **0.36 (0.22 to 0.50)**  **<0.001** | 0.30 (0.14 to 0.45)  <0.001 |
| Serum Insulin (mU/L) | 0.07 (-0.09 to 0.23)  0.375 | **0.24 (0.09 to 0.38)**  **0.001** | **0.19 (0.02 to 0.35)**  **0.026** | **0.19 (0.04 to 0.34)**  **0.016** | **0.29 (0.15 to 0.43)**  **<0.001** | 0.21 (0.05 to 0.36)  0.008 |
| DM Status |  |  |  |  |  |  |
| No | Ref. | Ref. | Ref. | Ref. | Ref. | Ref. |
| Pre-DM | **0.44 (0.06 to 0.82) (0.023)** | 0.22 ( -0.12 to 0.57)  0.202 | 0.18 (-0.17 to 0.53)  0.304 | 0.23 (-0.15 to 0.60)  0.234 | 0.24 (-0.10 to 0.59)  0.163 | 0.16 (-0.18 to 0.51)  0.349 |
| DM | **0.89 (0.52 to 1.25)**  **<0.001** | **0.70 (0.37 to 01.03) <0.001** | **0.63 (0.29 to 0.97) <0.001** | **1.11 (0.70 to 1.52)**  **<0.001** | **0.97 (0.58 to 1.35)**  **<0.001** | 0.85 (0.46 to 1.24) <0.001 |
| Oral Hypoglycemic agent use | **0.85 (0.45 to 1.25)**  **<0.001** | **0.73 (0.37 to 1.09)**  **<0.001** | **0.66 (0.29 to 1.03)**  **<0.001** | **1.08 (0.64 to 1.52) <0.001** | **0.90 (0.49 to 1.31) <0.001** | 0.82 (0.41 to 1.23)  <0.001 |
| Metabolic Syndrome | 0.91 (0.62 to 1.19)  <0.001 | 0.77 (0.51 to 1.03)  <0.001 | 0.76 (0.47 to 1.94)  <0.001 | 0.67 (0.33 to 1.01)  <0.001 | 0.60 (0.28 to 0.92)  <0.001 | 0.35 (-0.00 to 0.71)  0.053 |

Table S2. The sex-specific association of Log CAC with cumulative FBG exposure from exam 1 to exam 5 using Area Under Curve (AUC), and length of diagnosed DM and time points with high FBG, in total population. *Model 1 is adjusted for age; Model 2 is Model 1 plus race and BMI at exam 5. FBG, fasting blood glucose; DM, diabetes mellitus*

| *Variable* | *Population* | Beta (95%CI)  P value | | |
| --- | --- | --- | --- | --- |
|  |  | **Crude** | **Adjusted Model 1** | Adjusted Model 2 |
| *Cumulative FBG exposure* | *Male* | **0.08 (0.02 to 0.14)**  **0.009** | **0.07 (0.01 to 0.13)**  **0.013** | 0.06 (-0.00 to 0.11) 0.053 |
|  | *Female* | **0.11 (0.06 to 0.16)**  **<0.001** | **0.11 (0.04 to 0.13)**  **<0.001** | 0.08 (0.04 to 0.13)  <0.001 |
| *Time-points with high FBG* | *Male* | **0.19 (0.10 to 0.28)**  **<0.001** | **0.14 (0.05 to 0.22)**  **0.002** | 0.11 (0.02 to 0.19)  0.014 |
|  | *Female* | **0.25 (0.17 to 0.33)**  **<0.001** | **0.17 (0.10 to 0.25)**  **<0.001** | 0.16 (0.08 to 0.23)  <0.001 |
| Length of diagnosed DM |  |  |  |  |
| *Never* | *Male* | Ref. | Ref. | Ref. |
| *<5 yrs with DM* |  | 0.59 (-0.04 to 1.22)  0.067 | 0.55 (-0.03 to 1.14)  0.065 | 0.44 (-0.14 to 1.03) 0.137 |
| *>5 yrs with DM* |  | **1.29 (0.77 to 1.82)**  **<0.001** | **1.01 (0.52 to 1.50)**  **<0.001** | 0.94 (0.45 to 1.43)  <0.001 |
| *Never* | *Female* | Ref. | Ref. | Ref. |
| *<5 yrs with DM* |  | 0.06 (-0.44 to 0.57)  0.806 | 0.21 (-0.25 to 0.66)  0.377 | 0.11 (-0.36 to 0.57)  0.648 |
| *>5 yrs with DM* |  | 1.43 (0.94 to 1.91)  <0.001 | 1.04 (0.60 to 1.48)  <0.001 | 0.99 (0.54 to 1.43)  <0.001 |

Table S3. The association of Log CAC with categorized change in FBG (normal (FBG=<100 mg/dL and above normal FBG >100 mg/dL) from exam 4 to exam 5 (mean interval of 4.6 yrs), from exam 3 to exam 5 (mean interval of 6.3 yrs), and exam 2 to exam 5 (mean interval of 7.8 yrs). *Model 1 is adjusted for age; Model 2 is Model 1 plus race and BMI at exam 5. FBG, fasting blood glucose.*

|  | Female | | | Male | | |
| --- | --- | --- | --- | --- | --- | --- |
|  | **Beta (95% CI)**  **p value** | | | Beta (95% CI)  p value | | |
| Categories of Change in FBG | **Crude** | **Adjusted model 1** | **Adjusted model 2** | **Crude** | **Adjusted model 1** | Adjusted model 2 |
| Exam 2 to Exam 5 | | | | | | |
| *Sustained low* | Ref. | Ref. | Ref. | Ref. | Ref. | Ref. |
| *Decreased* | **1.00 (0.42 to 1.59) <0.001** | **0.57 (0.04 to 1.10) 0.036** | 0.48 (-0.06 to 1.01) 0.081 | 0.55 (-0.18 to 1.27)  0.138 | 0.01 (-0.67 to 0.69)  0.971 | -0.03 (-0.71 to 0.64) 0.919 |
| *Increased* | **0.44 (0.01 to 0.87)**  **0.047** | 0.28 (-0.11 to 0.67)  0.161 | 0.24 (-0.15 to 0.64)  0.227 | 0.29 (-0.17 to 0.75)  0.223 | 0.34 (-0.09 to 0.77)  0.121 | 0.28 (-0.15 to 0.71)  0.198 |
| *Sustained High* | **0.80 (0.42 to 1.18) <0.001** | **0.54 (0.19 to 0.89)**  **0.002** | **0.46 (0.10 to 0.82) 0.013** | **0.83 (0.41 to 1.25)**  **<0.001** | **0.68 (0.29 to 1.07)**  **<0.001** | 0.56 (0.16 to 0.95)  0.005 |
| Exam 3 to Exam 5 | | | | | | |
| *Sustained low* | Ref. | Ref. | Ref. | Ref. | Ref. | Ref. |
| *Decreased* | **0.88 (0.25 to 1.51)**  **0.006** | **0.63 (0.05 to 1.21)**  **0.032** | 0.56 (-0.02 to 1.14)  0.059 | 0.25 (-0.48 to 0.99)  0.494 | -0.35 (-1.03 to 0.34)  0.320 | -0.41 (-1.09 to 0.27)  0.239 |
| *Increased* | **0.52 (0.10 to 0.94)**  **0.015** | **0.40 (0.01 to 0.78)**  **0.043** | 0.36 (-0.03 to 0.75)  0.070 | 0.26 (-0.18 to 0.71)  0.249 | 0.26 (-0.15 to 0.68)  0.217 | 0.19 (-0.23 to 0.61) 0.375 |
| *Sustained High* | **0.70 (0.31 to 1.09) <0.001** | **0.44 (0.09 to 0.80)**  **0.014** | **0.37 (0.00 to 0.74)**  **0.047** | **0.81 (0.39 to 1.24)**  **<0.001** | **0.69 (0.30 to 1.09)**  **<0.001** | 0.57 (0.17 to 0.97)  0.005 |
| Exam 4 to Exam 5 | | | | | | |
| *Sustained low* | Ref. | Ref. | Ref. | Ref. | Ref. | Ref. |
| *Decreased* | **0.54 (0.02 to 1.07)**  **0.041** | 0.37 (-0.10 to 0.85)  0.123 | 0.32 (-0.16 to 0.80)  0.190 | 0.14 (-0.47 to 0.76)  0.651 | -0.20 (-0.77 to 0.37)  0.498 | -0.21 (-0.78 to 0.36)  0.472 |
| *Increased* | 0.02 (-0.46 to 0.50)  0.939 | -0.18 (-0.61 to 0.26)  0.427 | -0.22 (-0.66 to 0.22)  0.331 | 0.31 (-0.20 to 0.82)  0.228 | 0.41 (-0.06 to 0.88)  0.085 | 0.36 (-0.10 to 0.83)  0.127 |
| *Sustained High* | 0.94 (0.57 to 1.30)  <0.001 | 0.72 (0.39 to 1.06) <0.001 | 0.66 (0.32 to 1.00)  <0.001 | 0.66 (0.26 to 1.06)  0.001 | 0.53 (0.16 to 0.91)  0.005 | 0.42 (0.04 to 0.80)  0.028 |

Figure S1. Flowchart of the progression of participants.

Participants with measured CCC, available FBG and no history of bypass or cancer

N=2,305

Participants in the **repeated measure** analysis

N=2,092

Participants in the **cross-sectional** analysis

N=2,305

The MESArthritis Ancillary Study

N=3,305

Participants with measured CCC

N=2,562

Participants with unevaluable images due to artifacts and non-compliant FOV or reconstruction protocol

N=743

N=743

Participants with measured CCC and available FBG at exam 5

N=2,505

Participants with unavailable cross-sectional exam 5 data

N=57

Participants with history of coronary bypass surgery or cancer

N=200

Female participants

N=1,258

Male participants

N=1,047

Female participants

N=1,168

Male participants

N=924

Participants with no data inquiry in any of the 5 exams

N=215

Figure S2. Quantification of CCC in non-contrast cardiac CT scans using the VScore tool of the Vitrea software in three consequent slices (3mm thickness). *The calcified regions (HU>180) between the concave surface of medial end of the rib and sternum are marked (by green in right and by red in left side of the participant).*


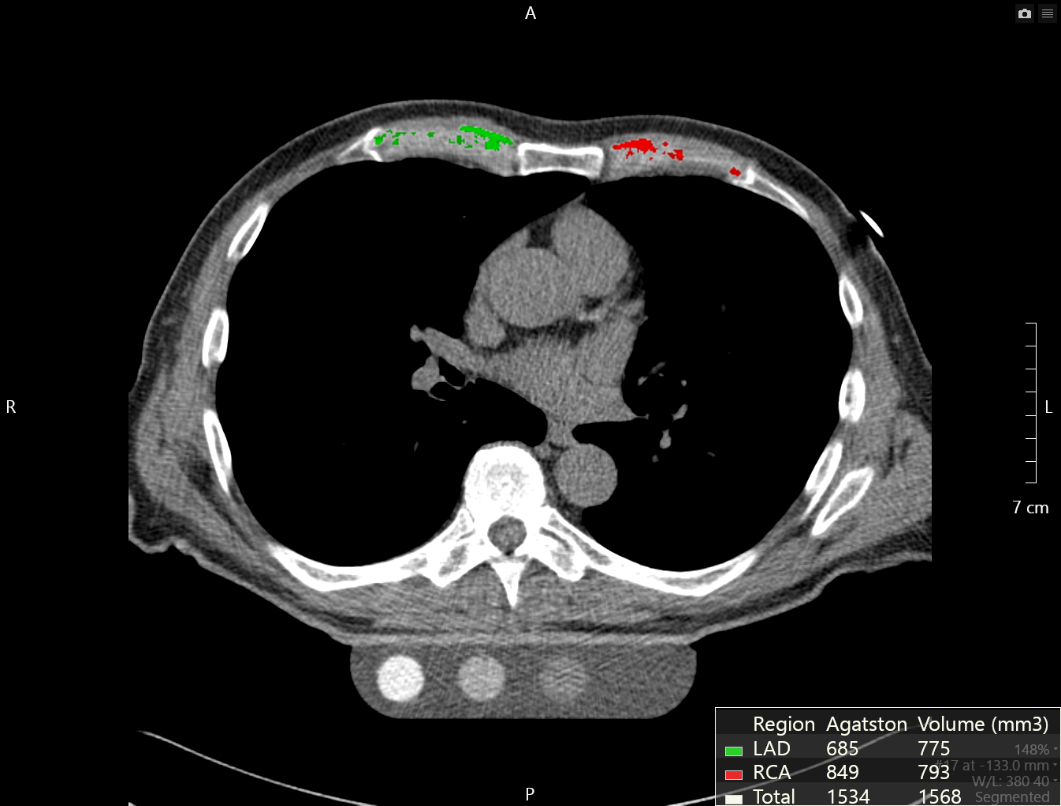

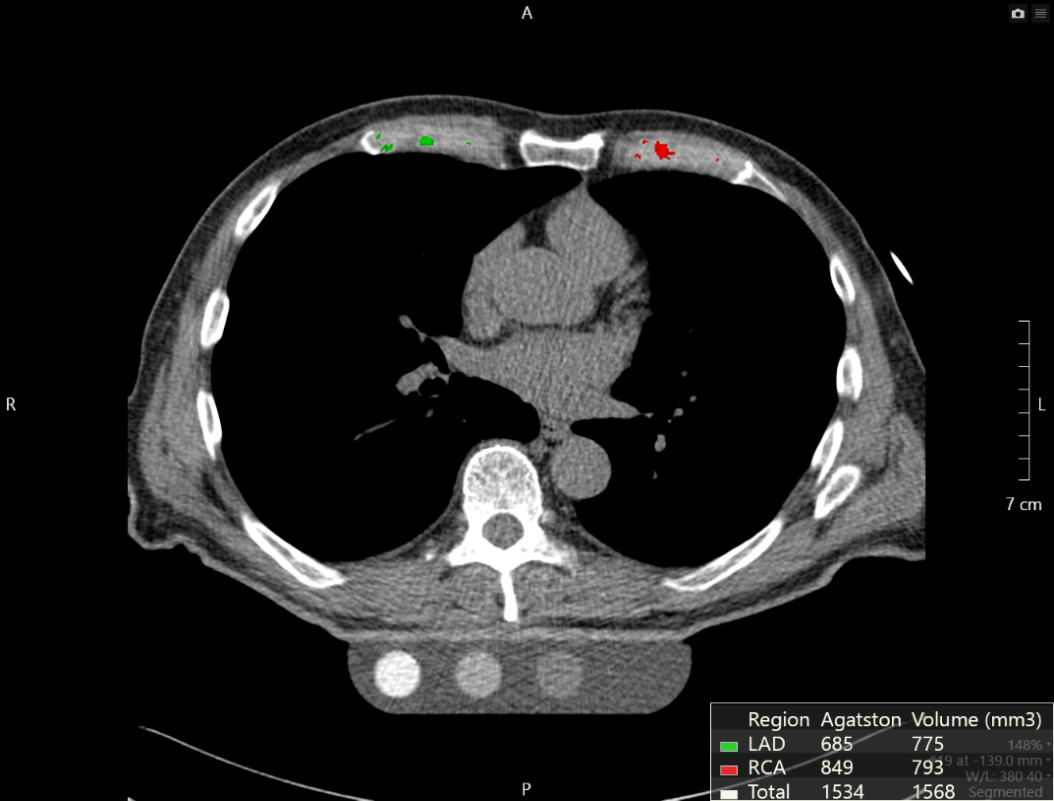

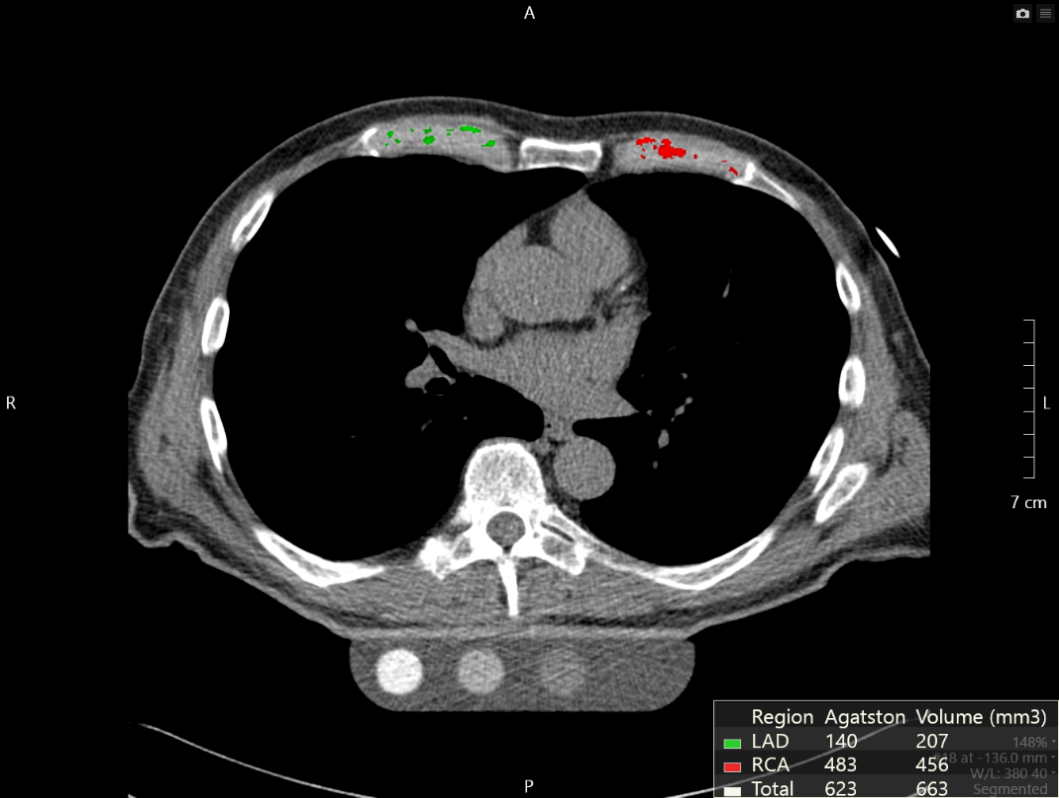

Supplement: Supplementary file 1 [file DataSheet_1.docx]
